# The scene of lung pathology during PRRSV-1 infection

**DOI:** 10.3389/fvets.2024.1330990

**Published:** 2024-03-19

**Authors:** Inés Ruedas-Torres, José María Sánchez-Carvajal, Francisco Javier Salguero, Francisco José Pallarés, Librado Carrasco, Enric Mateu, Jaime Gómez-Laguna, Irene Magdalena Rodríguez-Gómez

**Affiliations:** ^1^United Kingdom Health Security Agency (UKHSA Porton Down), Salisbury, United Kingdom; ^2^Department of Anatomy and Comparative Pathology and Toxicology, Pathology and Immunology Group (UCO-PIG), UIC Zoonosis y Enfermedades Emergentes ENZOEM, International Agrifood Campus of Excellence (CeiA3), Faculty of Veterinary Medicine, University of Córdoba, Córdoba, Spain; ^3^Department of Animal Health and Anatomy, Autonomous University of Barcelona, Barcelona, Spain

**Keywords:** PRRSV-1, pathology, lung, interstitial pneumonia, bronchopneumonia, inflammation, virulence

## Abstract

Porcine reproductive and respiratory syndrome (PRRS) is one of the most economically important infectious diseases for the pig industry worldwide. The disease was firstly reported in 1987 and became endemic in many countries. Since then, outbreaks caused by strains of high virulence have been reported several times in Asia, America and Europe. Interstitial pneumonia, microscopically characterised by thickened alveolar septa, is the hallmark lesion of PRRS. However, suppurative bronchopneumonia and proliferative and necrotising pneumonia are also observed, particularly when a virulent strain is involved. This raises the question of whether the infection by certain strains results in an overstimulation of the proinflammatory response and whether there is some degree of correlation between the strain involved and a particular pattern of lung injury. Thus, it is of interest to know how the inflammatory response is modulated in these cases due to the interplay between virus and host factors. This review provides an overview of the macroscopic, microscopic, and molecular pathology of PRRSV-1 strains in the lung, emphasising the differences between strains of different virulence.

## 1 Introduction

More than 30 years after its first description (1, 2), porcine reproductive and respiratory syndrome (PRRS) continues to be one of the greatest threats to the swine industry worldwide (3–6). It has been recently estimated that the economic losses attributable to PRRS in Europe are € 74,181 per farm per year, corresponding to an average of € 255 per sow per year (5). For the American swine industry, the disease costs about $ 664 million annually according to the study performed by Holtkamp et al. (6). Despite many attempts of the scientific community to develop an effective vaccine in the past decades, current available vaccines are not fully protective and only induce partial protection against heterologous strains (7).

The aetiological agent of this disease are PRRS viruses (PRRSV-1 and PRRSV-2), positive-single stranded RNA viruses classified within the genus *Betaarterivirus* (8, 9). The first European and American strains isolated at the beginning of the 90s, Lelystad (LV) strain and VR-2332 strain, respectively (2, 10), which displayed only about 60% of nucleotide similarity, led to the consideration of two different genotypes of the virus, named as European or genotype-1 (also known as PRRSV-1) and American or genotype-2 (also known as PRRSV-2) (11). Recently, the International Committee on Taxonomy of Viruses reclassified them as two different viral species, *Betaarterivirus suid-1* (PRRSV-1) and *Betaarterivirus suid-2* (PRRSV-2) (9). Four PRRSV-1 subtypes have been identified so far: the pan-European subtype-1, Eastern European subtypes-2 and 3, and subtype-4 with strains from Latvia and Belarus (12–15). PRRSV-2 is mostly prevalent in America and Asia with at least nine well-defined lineages (16). Following the isolation of Lena and SU1-bel strains, enhanced virulence of PRRSV-1 strains was rapidly associated to those included within subtype-3 (17, 18). However, other strains belonging to subtype-1, such as PR40, AUT15-33 and Rosalía, or subtype-2, such as BOR59 strain, showed also high virulence (19–23).

Clinical manifestations depend mostly on the virulence of the PRRSV-1 strain, although other factors, such as management practices, the immunological herd status, genetics of the pigs or co-infections are of importance. The main characteristics and differences in clinical signs, lesions, tropism, and immunological parameters between classic (moderately virulent) and highly virulent PRRSV strains have been recently reviewed (24). Moderately virulent PRRSV-1 strains are often involved in outbreaks of reproductive failure in sows and respiratory disease in growing pigs (2, 10). The manifestations of reproductive failure may vary from sporadic abortions to abortion storms, mainly in the third trimester of gestation, together with premature parturition, delivery of stillborn piglets, mummified piglets or weak-born piglets (2, 10, 18). In piglets of all ages, the virus targets the alveolar macrophages, producing some degree of interstitial pneumonia and impacting secondarily on the body weight gain (19, 25, 26). Experimental infections have shown that moderately virulent PRRSV-1-infected animals usually show lethargy, mild fever and mild to moderate respiratory signs, such as slight dyspnoea, but they usually fully recover after a few days (17–19, 21, 26, 27).

When a virulent PRRSV-1 strain is causing the infection, the clinical pattern changes substantially. High mortality (>20%), prolonged high fever (≥41°C) and severe respiratory disease are common findings of all infections with virulent PRRSV-1 strains (17, 19–21, 24, 25, 27–30). Cyanosis on the ears and tail, conjunctival hyperaemia, and diarrhoea are also clinical manifestations observed in pigs infected with virulent PRRSV-1 strains (19–21, 30). Moreover, clinical manifestations of virulent PRRSV-1 strains appear early after infection, even after 1 day, with a rapid disease onset (18, 19, 21, 27).

The emergence of these virulent PRRSV-1 strains during the last two decades in Europe, such as Lena and SU1-bel strains in Belarus (18), PR40 strain in Italy (19) or Rosalía in Spain (22, 23), has gained special concerns within the pig industry and the research community, due to the high morbidity and mortality rates as well as the severity of the lesions, mainly in the lung (18, 19). Additionally, particular attention has been given to the potential mutation rate and recombination among endemic and emerging strains which may lead to a scenario with the appearance of potentially devastating outbreaks, such as the one caused by Rosalía strain in Spain in 2020, which resulted from the recombination of different PRRSV-1 isolates (22, 23).

PRRSV possesses a restricted cell tropism for CD163^+^ cells (31, 32). CD163 is mainly expressed on cells of the monocyte/macrophage lineage, especially the pulmonary alveolar macrophage (PAM), making the lung its main target organ (33). This review discusses in depth the pathology of PRRS in the lung, emphasising the different patterns of lung injury observed among PRRSV-1 strains and the possible interaction between the virus and host factors.

## 2 PRRSV-1 lung lesions

### 2.1 Macroscopic lung lesions after PRRSV-1 infection

#### 2.1.1 Interstitial pneumonia as PRRSV-induced gross lung lesion

Moderately virulent PRRSV-1-infected grower pigs usually show mild lesions in the lung which frequently go unnoticed, but in the worst-case scenario, pigs develop mild to moderate interstitial pneumonia (2, 10, 29).

**Figure 1:**
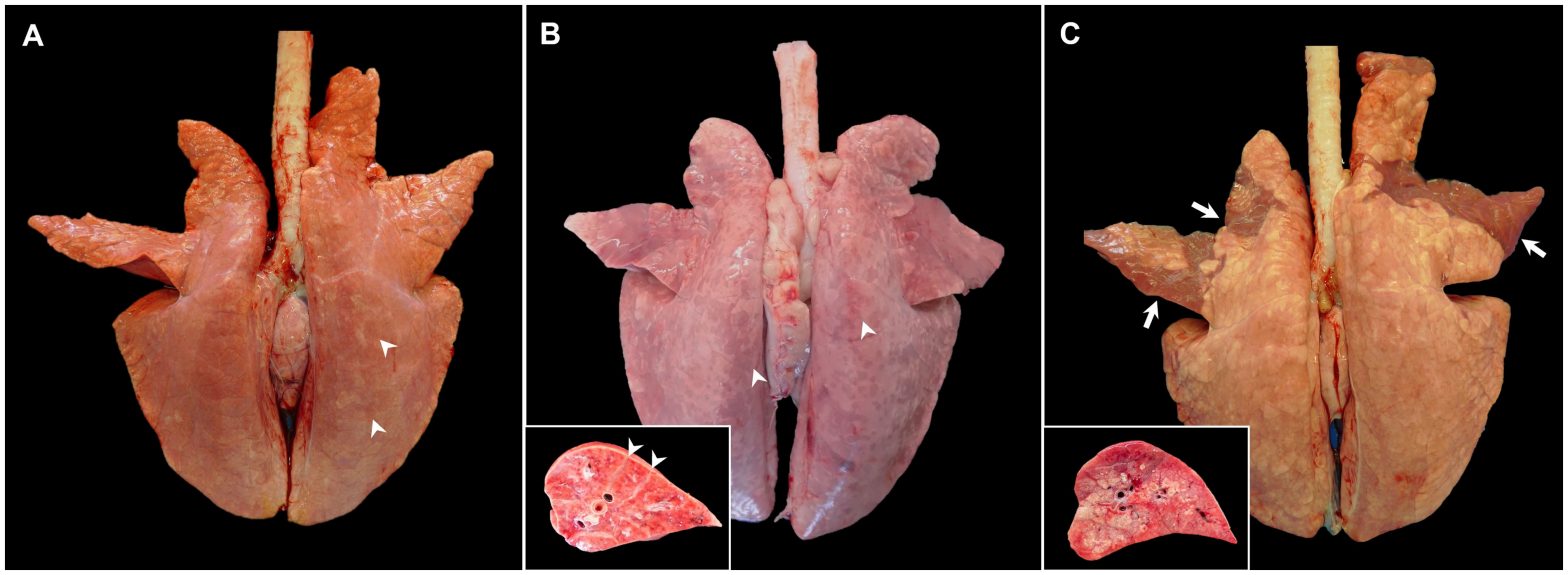
Gross pictures of lungs from pigs experimentally infected with PRRSV-1 strains of different virulence and euthanised at 8–10 dpi. **(A)** Lung of a pig infected with 3249 strain showing tan mottling areas (arrowheads), and not collapsing after removal from thoracic cavity. **(B)** Lung from a pig infected with the highly virulent Rosalía strain displaying a marked reddish mottle pattern and diffuse firmness, especially in the dorsal aspect of the lung (arrowheads). Inset shows higher magnification and section of one of the affected lobes. Arrowheads show interstitial oedema. **(C)** Lung from a Lena-infected pig exhibiting tan areas and rubbery texture but also patchy ventral areas of consolidation of the cranial and middle lung lobes (arrows). Inset shows higher magnification and section of the consolidation area of one of the affected lobes.

The key macroscopic finding in moderately virulent PRRSV-1-infected animals is interstitial pneumonia that macroscopically is characterised by a mottled tan to red and rubbery pulmonary parenchyma which fails to collapse after opening the thoracic cavity, mainly visible at the caudal lobe, (Figure 1A, arrowheads), which can be particularly severe in cases of virulent strains, such as Rosalía strain (Figure 1B, arrowheads) (2, 10, 17, 19, 25–28, 34, 35). The macroscopic scoring system developed by Halbur et al. (36) has been frequently used to evaluate the severity and distribution of pulmonary lesions in PRRS, which considers each lung lobe at the dorsal and ventral view within the entire lung parenchyma (17, 25–28, 34, 37). According to the study performed by Morgan et al. (17) in pigs infected with the prototypical PRRSV-1 strain LV, interstitial pneumonia is visible from day 7 post-infection (pi) onwards; however, no gross lesions are usually detected at 1 month pi in these infected animals (17, 28, 34, 38). Gradual increase of lung consistency on cranial and middle lobes with the progression of the disease over time has been reported, indicating foci of consolidation due to secondary bacterial infections (27, 37). Macroscopically, consolidated areas were swollen, firm and reddish, and clearly demarcated from the rest of the lung. At sectioning, mucopurulent exudate could be observed on airways sometimes, all of it indicative of suppurative bronchopneumonia (39).

#### 2.1.2 Suppurative bronchopneumonia as accompanying lesion to interstitial pneumonia in virulent PRRSV-1-infected animals

In general, field PRRSV infections are frequently accompanied by secondary bacterial complications, conveying to suppurative bronchopneumonia. However, when a virulent PRRSV-1 strain infects the pig under experimental conditions, gross lung lesions are more marked, showing severe diffuse interstitial pneumonia which, different to moderately virulent PRRSV-1 strains, is more commonly accompanied by foci of consolidation from very early stages of infection, resulting from suppurative bronchopneumonia in cranioventral areas (Figure 1C, arrows and inset) (18–20, 25, 27, 28, 30, 34, 37). According to different experimental studies, in infections caused by virulent strains, suppurative bronchopneumonia is already present at very early time points (3 days pi, dpi) and usually culminates between the first and the second-week pi (wpi), exhibiting higher gross pathology scores than the lungs from moderately virulent PRRSV-1-infected animals according to the scoring system developed by Halbur et al. (17, 27, 34, 38). For example, in the study performed by Morgan et al. (17), lungs from animals infected with the virulent SU1-bel strain showed more than 20 score points of difference than those infected with LV strain at 7 dpi, due to secondary bronchopneumonia (17). Interestingly, gross lesions were not visible in the lungs of SU1-bel-infected piglets at 1-month pi (17) and were not observed either in the lung of Lena-infected piglets (38). On the contrary, Frydas et al. (37) showed a similar gross lesion percentage between the low-virulent PRRSV-1 07V063 strain and the 13V091 strain, considered as a virulent PRRSV-1 strain (subtype-1) (37). The discrepancies among these and other studies could be associated to the differences between each experimental set-up, including the dose and route of infection, the inoculum passage and volume, the age of the pigs and their genetic background. Lung weight relative to body weight is a potential indicator of lung inflammation and was used by Weesendorp et al. (40) to evidence differences between lungs from Lena- and LV-infected animals. Lungs from Lena-infected pigs showed higher relative lung weight in comparison with those from LV-infected pigs and uninfected control animals at 1 wpi (40). This relative lung weight decreased drastically at 46 dpi in the Lena-infected group (40), which could indicate a partial resolution of the induced pulmonary lesion.

Although bronchopneumonia is associated with the presence of pathogenic bacteria, their isolation has not been always demonstrated when present in PRRSV-infected animals. Severe lung consolidation was reported in animals infected with 07 V063 and 13 V091 strains, however, no specific bacterial pathogens were isolated (37). On the other hand, the participation of *Staphylococcus hyicus* was demonstrated by conventional bacterial culture in an outbreak that took place in Austria in 2015, caused by the virulent strain AUT15-33, in animals that showed suppurative bronchopneumonia as well as porcine circovirus type 2 (PCV2) coinfection (20). Other macroscopic lung lesions have been described in the lung of virulent PRRSV-1-infected animals. For instance, Karniychuk et al. (18) reported fibrinous pleuropneumonia in 7 out of 10 pigs infected with the virulent PRRSV-1 Lena strain. *Arcanobacterium pyogenes* (currently *Trueperella pyogenes*) and *Streptococcus suis* were isolated in 2 of these animals, whereas no viruses, including PCV2 or swine influenza virus (SIV) were detected (18). Pleurisy was also observed in 2 out of 8 Lena-infected piglets in the study published by Renson et al. (29). Gross lesions secondary to interstitial pneumonia and bronchopneumonia such as multifocal to coalescing areas of atelectasis, congestion, and interstitial and alveolar oedema have also been described in other virulent PRRSV-1 infections (19). The disturbance of the physical barriers and immune response by several viruses, such as PRRSV, PCV2 or SIV, and *Mycoplasma hyopneumoniae*, among others, which are primary agents of the porcine respiratory disease complex (PRDC), is plausible to play a role in the coinfection with secondary endemic bacteria (*Pasteurella multocida*, *Bordetella bronchiseptica*, *Gläesserella parasuis*, etc.) (41, 42) or the proliferation of lung commensal microorganisms. Thus, further studies should address a proper characterisation of the pathogenic bacteria involved in the pathogenesis of the bronchopneumonia that is frequently observed concomitantly in virulent PRRSV-1 infections under experimental and field conditions.

### 2.2 Microscopic lesions induced by PRRSV-1 strains

#### 2.2.1 Histopathological features of PRRSV-induced interstitial pneumonia

**Figure 2:**
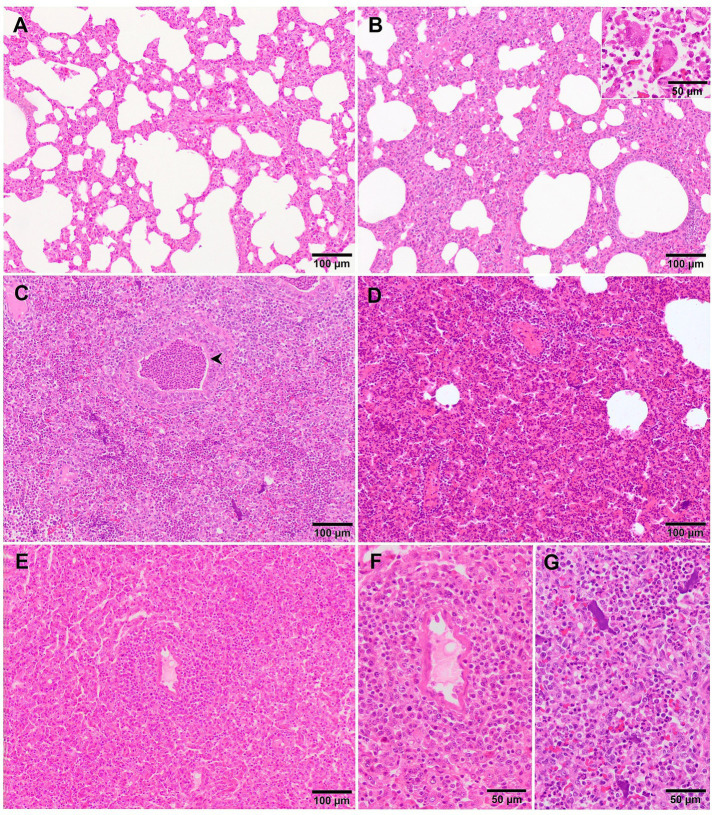
Microscopic pictures of the lung of representative pigs experimentally infected with PRRSV-1 strains of different virulence and euthanised at 8–10 dpi. **(A)** Mild thickening of the alveolar septa because of minimal infiltration of macrophages and lymphocytes in the lung tissue of a piglet infected with 3249 strain. **(B)** Moderate to severe thickening of the alveolar septa due to marked infiltration of mononuclear cells with the presence of a syncytia (inset) in the lung of a Lena-infected pig. **(C)** Lung tissue of a Lena-infected pig showing, together with thickening of the alveolar septa, degenerated neutrophils within the lumen of bronchioles (arrowhead) and alveoli as well as cellular debris and aggregates of free chromatin (see for details “**G**”). **(D)** Moderate thickening of alveolar septa with characteristic perivascular lymphocytic and histiocytic infiltrate together with areas of moderate atelectasis in the lung of SU1-bel-infected pig. **(E)** Similar lesions as reported in “**D**” with marked infiltration of macrophages in the alveolar septa and atelectasis in the lung of a pig infected with Rosalía strain. See “**F**” for detail of the periarteriolar infiltrate.

**Figure 3:**
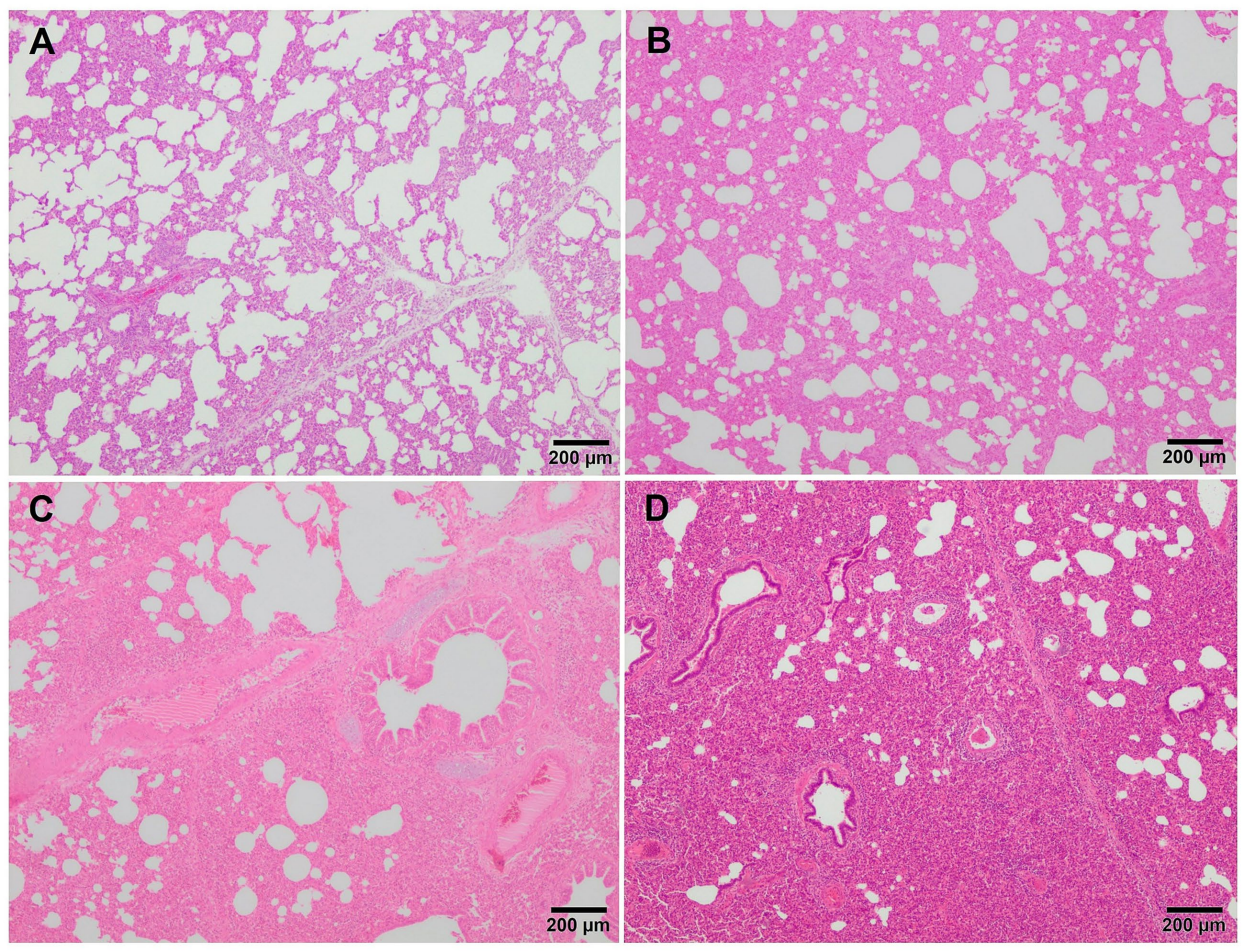
Microscopic pictures of representative score of interstitial pneumonia in PRRSV-1 infected pigs. **(A)** Score 1, mild interstitial pneumonia. Mild thickening of the alveolar septa because of minimal infiltration of macrophages and lymphocytes in the lung tissue of a piglet infected with 3249 strain. **(B)** Score 2, moderate interstitial pneumonia. Thickening of the alveolar walls due to moderate infiltration of macrophages and lymphocytes in the lung tissue of a piglet infected with the virulent Lena strain. **(C)** Score 3, moderate diffuse interstitial pneumonia. Infiltration of macrophages and scattered lymphocytes in the lung tissue of a piglet infected with the virulent Lena strain. **(D)** Score 4, severe diffuse interstitial pneumonia in the lung tissue of a piglet infected with the virulent Lena strain.

Microscopically, interstitial pneumonia has been reported as the distinctive lesion during PRRS, characterised by multifocal hypertrophy and hyperplasia of type II pneumocytes and alveolar septa thickening with infiltration of mononuclear cells, mainly lymphocytes and macrophages (36, 39, 43). Macrophage alveolar exudation is usually present and hyperplastic pneumocytes may form a continuous layer of cuboidal epithelium lining the alveolus (39, 44). Typically, the bronchiolar epithelium from PRRSV-infected animals is not affected, a finding that could point into the direction of other viral infections, such as SIV (39). Interstitial pneumonia induced by PCV2 shows a granulomatous pattern with syncytial cells, lymphocytes and polymorphonuclear cells infiltrating the alveolar septa (39, 44). Nonetheless, these findings are not always evident and a clear distinction between PCV2 and PRRSV infection may be hard to find. In PRRSV-1 uncomplicated cases, a mild to moderate multifocal to extensive interstitial pneumonia is usually observed (Figure 2A), which increases in severity alongside the virulence of the strain, and occasionally finding syncytia in those cases too (Figure 2B, inset) (17, 19, 21, 27, 38). Halbur et al. (36) described a scoring system to evaluate interstitial pneumonia which is widely used in porcine pathology studies and for both PRRSV species (27, 28, 34). Briefly, no microscopic lesion is scored as 0, mild interstitial pneumonia is scored as 1 (Figure 3A), moderate multifocal interstitial pneumonia is scored as 2 (Figure 3B), moderate diffuse interstitial pneumonia is scored as 3 (Figure 3C), and severe interstitial pneumonia is scored as 4 (Figure 3D) (36). A separate evaluation of each lung lobe, cranial, middle, and caudal is highly recommended to evaluate the distribution of the lesions as well as to avoid misinterpretation of lung lobes affected by bronchopneumonia. According to this scoring system, lungs from animals infected with virulent PRRSV-1 strains such as SU1-bel or Lena strains, displayed the most severe lesions in comparison with the low-virulent strains used in different experimental trials after 1 wpi (27, 28, 34).

#### 2.2.2 Histopathological features of suppurative bronchopneumonia in virulent PRRSV-1 strains

**Figure 4:**
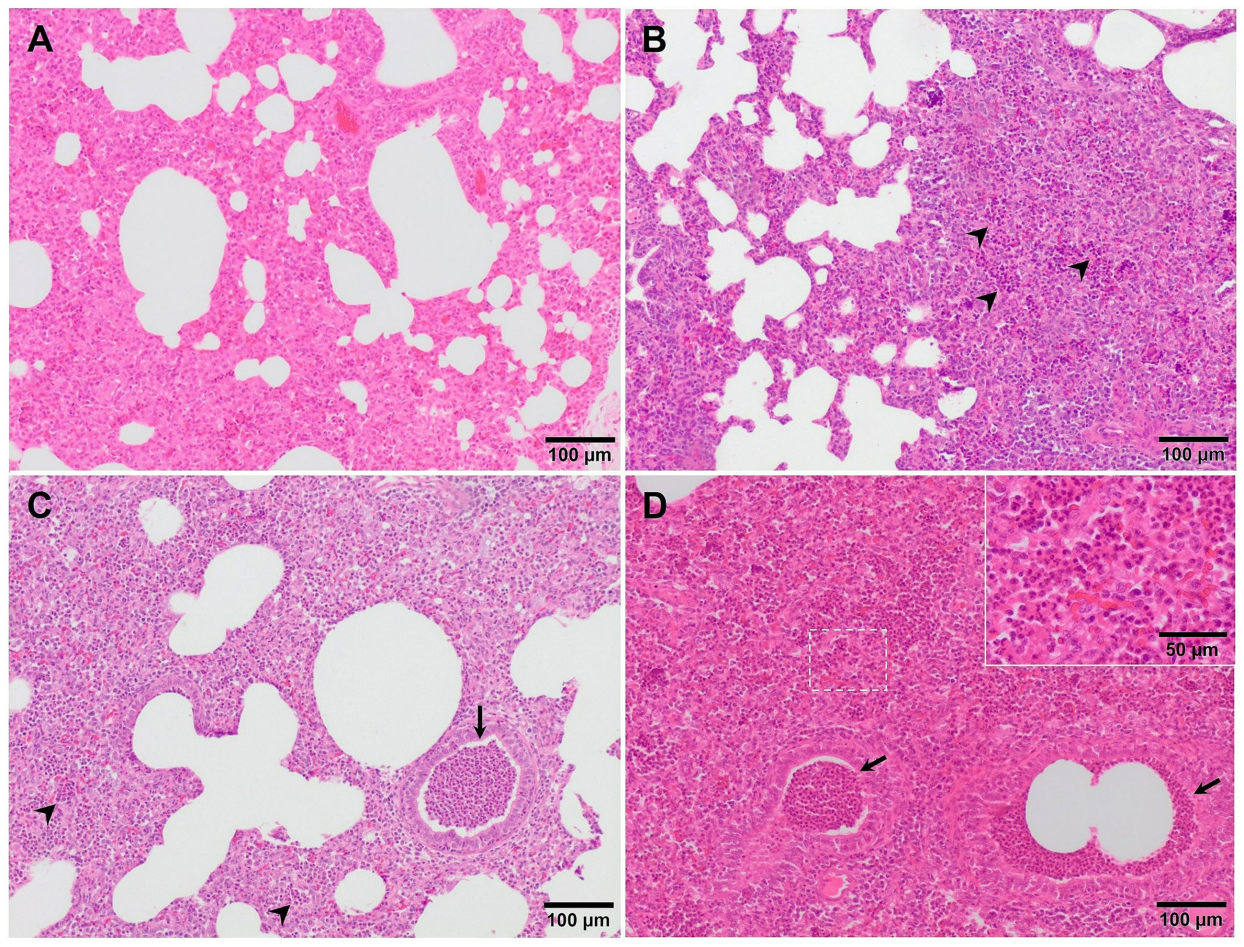
Microscopic pictures of representative score of suppurative bronchopneumonia in PRRSV-1 infected pigs. **(A)** Score 1, mild bronchopneumonia in the lung from 3249 infected animal. Granulocytes and macrophages are present in the alveolar septa. **(B)** Score 2, moderate multifocal bronchopneumonia in the lung from an animal infected with the virulent Lena strain. A high number of granulocytes (arrowheads) and macrophages, together with cell debris infiltrate the alveolar walls. **(C)** Score 3, moderate diffuse bronchopneumonia in the lung from an animal infected with Lena strain. Granulocytes (arrowheads), macrophages, and cellular debris within the lumen of bronchi, bronchioles (arrow), and alveoli. Inset show infiltration of macrophages and scattered lymphocytes and granulocytes. **(D)** Score 4, severe bronchopneumonia in the lung from an animal infected with Lena strain. Arrowheads and inset show infiltration of granulocytes within bronchioli and alveoli, respectively.

**Figure 5:**
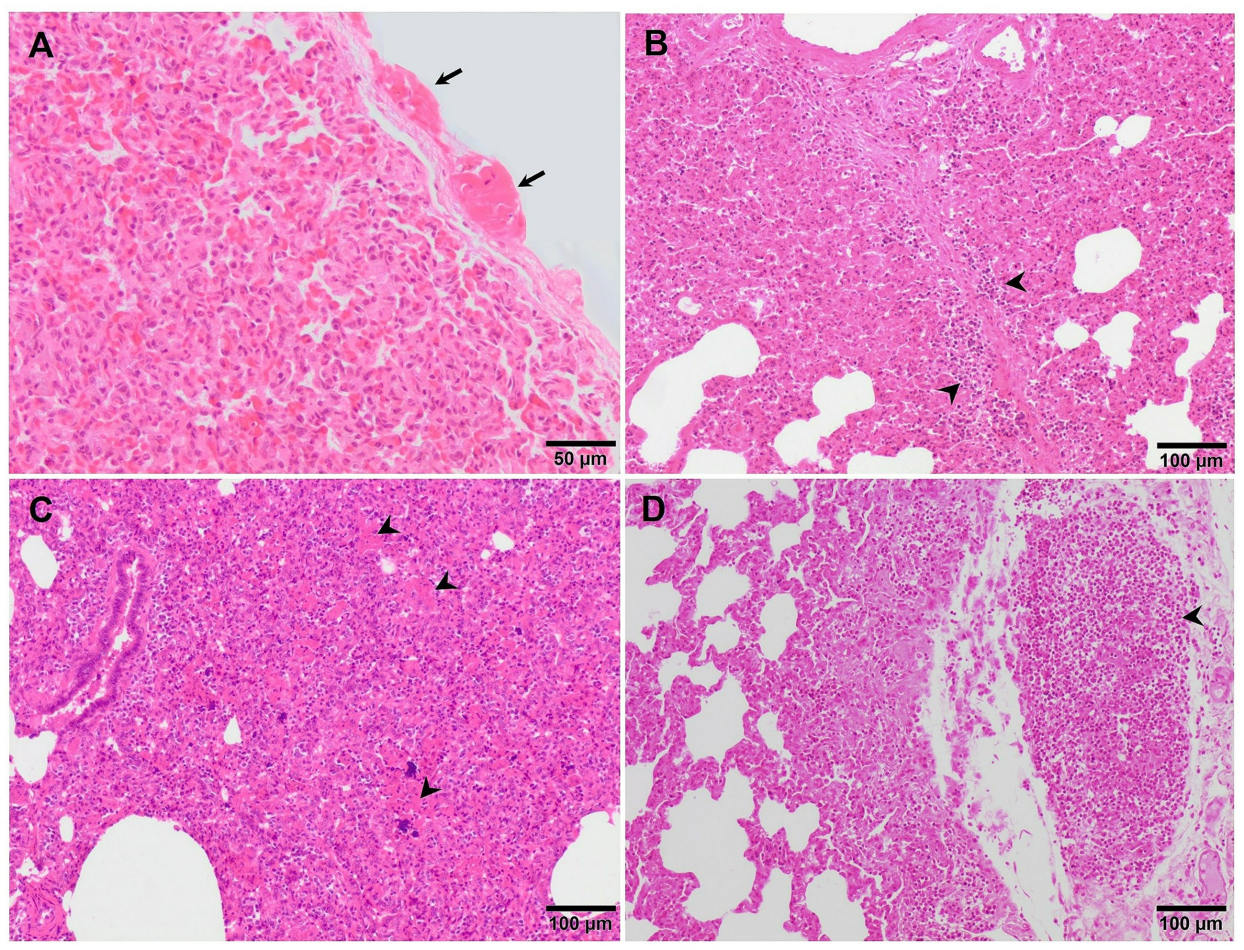
Microscopic pictures of the lung of representative pigs experimentally infected with the virulent Lena strain euthanised at 8 dpi **(A)** and with the highly virulent Rosalía strain and euthanised at 10 **(B,C)** and 35 dpi **(D)**. **(A)** Thickening of the pleura due to the presence of fibrin (fibrinous pleuritis). **(B)** Alveolar septa are thickened by macrophages and lymphocytes which also predominantly infiltrate the interlobular septa (arrowheads). **(C)** The lung is moderately atelectatic, with type II pneumocytes hyperplasia and alveoli filling in by necrotic cellular debris (arrowheads), including basophilic clumps of chromatin, compatible with proliferative and necrotising areas of pneumonia. **(D)** Subpleural well-demarcated accumulation of lymphocytes consistent with a tertiary lymphoid organ.

Although, interstitial pneumonia is the hallmark of PRRSV infection, the presence and proliferation of specific commensal pathogens from the lung microbiome together with secondary bacterial infections cause suppurative bronchopneumonia, frequently found in virulent PRRSV-infected animals (Figure 2C) (20, 25, 27). Suppurative bronchopneumonia is characterised by abundant granulocytes, macrophages, and cellular debris within the lumen of bronchi, bronchioles, and alveoli (39). This made it necessary to create a scoring system to evaluate this lesion, if present (27). The score system estimates the severity and distribution of the suppurative bronchopneumonia as follows: 0, no microscopic lesions; 1, mild bronchopneumonia (Figure 4A); 2, moderate multifocal bronchopneumonia (Figure 4B); 3, moderate diffuse bronchopneumonia (Figure 4C); and 4, severe bronchopneumonia (Figure 4D). With this scoring system, lung sections from pigs infected with the virulent PRRSV-1 Lena strain showed a score of 1 or 2 at 6 dpi, whereas animals infected with the moderately virulent 3249 strain, reached these scores 1 week later (13 dpi) (27). This suppurative bronchopneumonia is usually accompanied by secondary atelectasis, oedema of the interlobular septa and dilation of lymphatic vessels (20, 27). The presence of fibrinous material in the pleura has been also described as a finding related to infection of the animals with the virulent PRRSV-1 Lena strain (Figure 5A) (18, 27).

**Figure 6:**
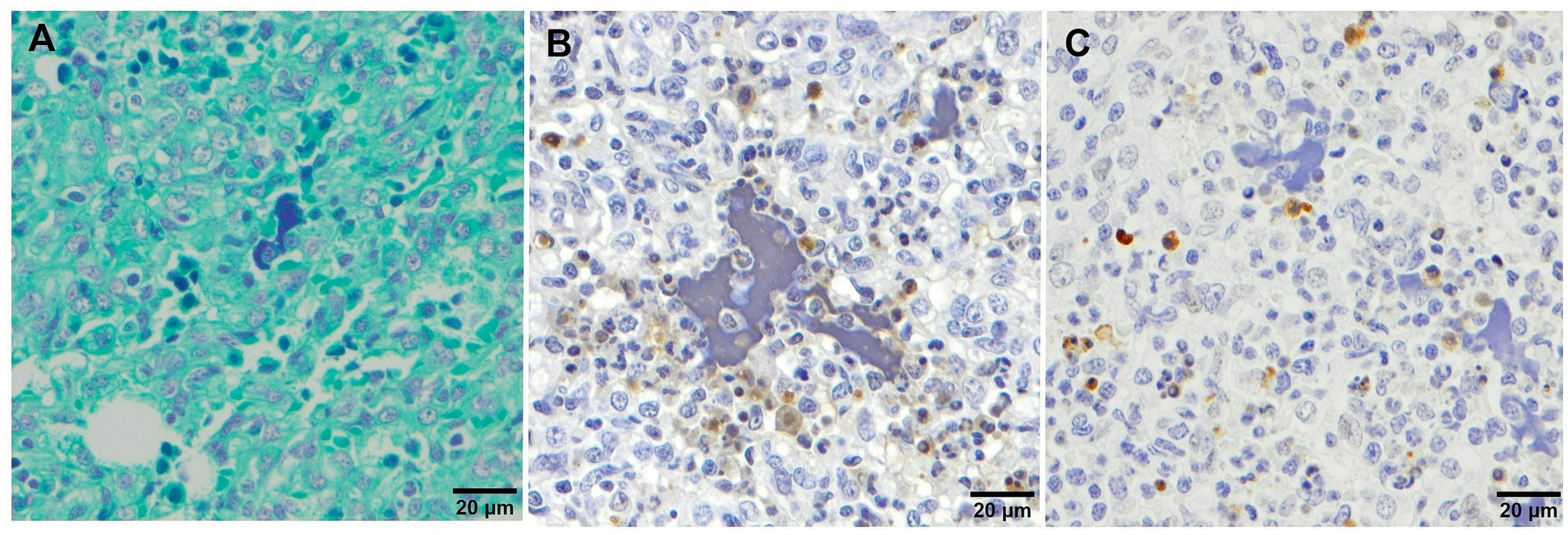
Microscopic pictures of clumps of free chromatin demonstrated using Feulgen staining **(A)**, TUNEL **(B)** and cleaved-caspase-3 **(C)** immunohistochemical staining in the lung tissue from virulent Lena infected animals with severe bronchopneumonia. **(A)** Feulgen^+^ staining which demonstrates the presence of clumps of free chromatin. **(B)** TUNEL and **(C)** cleaved-caspase-3 stainings showing the negativity of the clumps of free chromatin.

Multifocal pyknosis and presence of cellular debris in the septal interstitium and within alveoli have been described in piglets infected with some virulent PRRSV-1 strains (25, 27, 28) in association with regulated cell death (28). The presence of clumps of free chromatin (Figures 2C,G) demonstrated using Feulgen staining (Feulgen^+^) was frequently observed in Lena- and Rosalía-infected animals with the highest bronchopneumonia scores (27) (Figure 6A). Moreover, this amorphous material was identified as TUNEL^−^, a technique to detect DNA fragmentation (Figure 6B) and cleaved-caspase-3^−^ (executioner caspase, main marker of apoptosis) (Figure 6C), suggesting that these clumps may be associated with neutrophil extracellular traps (NETs) triggered within foci of suppurative bronchopneumonia (45). NETs formation in the context of virulent PRRSV strains might play a role either preventing microorganisms spread or favouring bacterial growth (45).

#### 2.2.3 Kinetics of microscopic changes in the lung of PRRSV-1 infected animals

Whereas in animals experimentally infected with moderately virulent PRRSV strains lesions are noticeable around the first wpi (27, 28, 34), in those infected with virulent PRRSV-1 strains microscopical lesions develop from 3 dpi onwards, reaching the maximum scores between the first and second wpi (27, 28, 34, 38). At 1-month after infection, interstitial pneumonia is only occasionally present and is of mild intensity (28, 34, 38). Balka et al. (25) observed a significant decrease in the presence of intra-alveolar cellular debris and in the number of intra-alveolar neutrophils throughout the course of the infection (from 10 to 21 dpi) with the virulent German 205817 strain together with an extensive type II pneumocyte proliferation, which was associated with the resolution of the lesion (25). In this sense, type II pneumocytes have been poorly characterised along PRRSV infection and might represent a target cell to understand the progression of the pathogenesis of this disease.

#### 2.2.4 Other microscopic lung lesional patterns associated with PRRSV-1 infection

Depending on the intensity of the pathological process, additional prominent lesions may be observed, such as proliferative and necrotising pneumonia (PNP), extensive areas of haemorrhage, and varying degrees of vasculitis, characterised by a prominent perivascular mononuclear infiltrate (19, 34, 46, 47). PNP is a severe form of interstitial pneumonia, characterised by two main histological features: (i) lymphohistiocytic interstitial inflammation with hypertrophy and proliferation of type II pneumocytes and (ii) presence of clumps of necrotic inflammatory cells within the alveolar spaces (44, 46, 48, 49). In a recent experimental study, several piglets infected with Rosalía strain developed PNP lesions as soon as 10 dpi (Figures 5B,C) (50). However, besides PRRSV, other aetiological agents such as PCV2 or SIV are usually involved in this lesional pattern (44, 46, 48, 49).

Additionally, other lesional patterns have been described in experimental studies performed with virulent PRRSV-1 strains. For example, Stadejek et al. (21) described a “honeycomb” pattern mostly in animals infected with the virulent PRRSV-1 strain BOR59, isolated in Belarus in 2009. This lesion, observed in animals euthanised at 17- and 22-dpi consisted of areas of fibroblast proliferation and fibrosis of the lung parenchyma, which gave the lung an appearance of loss of its structure (21). Additionally, in these infected animals, a high number of eosinophils, which were sometimes degranulated, was mainly observed in areas of severe lung fibrosis and around blood vessels (21). Associated with these lesions, hyperplasia of lymphoid follicles was also described, being especially noticeable in those animals infected with the virulent PRRSV-1 BOR59 strain in comparison with the moderately virulent strains evaluated: 18794 and ILI6 (21). Similarly, Weesendorp et al. (38), described a higher peribronchiolar cell infiltrate score, mainly formed by macrophage and monocytes at 7 dpi in lungs from animals infected with virulent PRRSV-1 Lena strain compared to the two other moderately virulent strains used in their study. However, this lesion was not so patent in the study performed with the same strain by Rodríguez-Gómez et al. (27), which could be due to the differences on the experimental design between both studies, such as the infectious dose or the age of the animals. A perivascular pattern of inflammatory cells, mainly lymphohistiocytic, was observed in lungs of animals infected with other virulent PRRSV-1 strains such as Lena, SU1-bel and Rosalía (Figures 2D–F) from 3 dpi onwards, being specially marked and obvious in lungs from Rosalía-infected pigs. However, this finding has been also observed from 8 dpi onwards in moderately virulent strains, like 3249 strain (25, 34, 45, 50). Interestingly, and different to what has been previously described for virulent strains, tertiary lymphoid organs were frequently observed in the lungs of Rosalía-infected piglets at 35 dpi (Figure 5D) (50). These structures have been related to robust immune responses to local inflammation at sites of tissue injury (51), indicating an ongoing pulmonary process, far from what has been described for other virulent PRRSV-1 strains in which after 1 month pi, the resolution of the pneumonia was taking place (25, 40).

## 3 Pathogenic mechanisms of pulmonary lesion in PRRS

### 3.1 Role of macrophages

**Figure 7:**
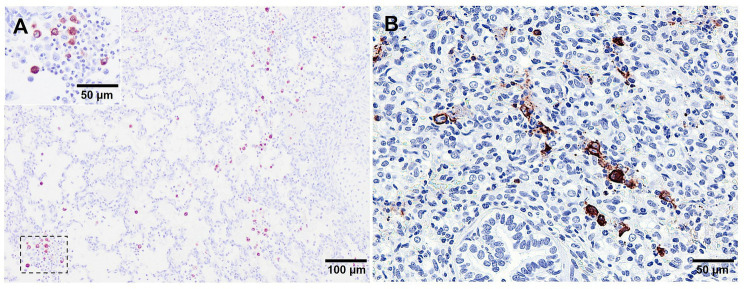
Microscopic pictures of PRRSV-N-protein immunohistochemical staining in lung tissue from virulent Lena infected animals euthanised at 8 dpi. **(A)** PRRSV-N-protein^+^ alveolar macrophages in a field of representative interstitial pneumonia. Inset shows higher magnification of PRRSV-N-protein^+^ alveolar macrophages. **(B)** Clusters of PRRSV-N-protein^+^ macrophages surrounded by apoptotic bodies.

The lung mononuclear phagocytic system comprises PAMs, interstitial lung macrophages, and, in several species including pigs, pulmonary intravascular macrophages (PIMs) (52). Whereas the primary function of PAMs is to establish a first line of phagocytic defence against microbial infections, septal macrophages (interstitial macrophages and PIMs) are more specialised in the release of proinflammatory cytokines that contribute to regulate pulmonary homeostasis (26, 53–55). PAMs are the primary target cells of PRRSV, although PIMs and interstitial macrophages are also susceptible to the infection (26, 33, 56). In this sense, immunolabelling of PRRSV-N-protein is mainly observed in PAMs and to a lesser extent in PIMs and interstitial macrophages (Figure 7A), with clusters of PRRSV-N-protein^+^ macrophages surrounded by apoptotic bodies within areas of bronchopneumonia in piglets infected with virulent strains (Figure 7B). PAMs express high levels of the CD163 scavenger receptor (56, 57), which plays a crucial role in PRRSV internalisation and disassembly by interacting with GP2 and GP4 viral proteins (32, 58, 59). Replication of PRRSV in PAMs, PIMs and interstitial macrophages leads to an impairment in their fundamental functions including: (a) phagocytosis, which is influenced by the interaction between the virus and CD169 receptor, (b) antigen presentation, and (c) production of proinflammatory cytokines (54, 60–64).

Furthermore, during infection, there is also early cell death of infected PAMs, as well as necrosis and apoptosis of other macrophages and lymphocytes in the lung and lymphoid organs (45, 65–68). These changes contribute to induce an imbalance in the pulmonary immune homeostasis of PRRSV-1-infected piglets, making them more susceptible to a wide range of respiratory pathogens, both viral and bacterial (69), leading to increased severity of clinical signs and pulmonary lesions in co-infected piglets (61).

In this sense, a significant depletion in the frequency of pulmonary CD163^+^ cells has been reported in PAMs and lung tissue sections of piglets infected with virulent strains such as Lena or SU1-bel around 7–10 dpi (27, 29, 45, 70, 71). CD163^+^ macrophages play a crucial role in tackling bacterial infections due to the sensing function of this molecule (72). Therefore, a reduction in the population of pulmonary CD163^+^ cells could potentially compromise lung phagocytic function, complicating cell debris clearance (29). This scenario could potentially create a favourable environment for co-infection with secondary commensal microorganisms, contributing to the development of suppurative bronchopneumonia. This phenomenon, resulting from the direct cytopathic effect of the virus on its target cells and the induction of regulated cell death in both infected and non-infected cells, has been extensively observed in the lungs and lymphoid organs of piglets infected with virulent PRRSV-1 strains (17, 45, 67).

On the other hand, a replenishment of CD163^+^ cells in the lung of SU1-bel infected pigs at 1 month after infection or at 2 wpi from Lena-infected piglets have been observed. This recovery of CD163^+^ PAMs has been reported parallel to an increase of arginase1^+^ (Arg1) macrophages (45), a common feature of M2-macrophages (73), suggesting a role in tissue repair, accelerating the resolution of inflammation (57, 74), which will be in accordance to what was observed during macroscopic lung examination in other studies (25, 40).

Considering *in vitro* studies using monocyte-derived macrophages (MDMs) and supported by the high functional plasticity of pulmonary macrophages and their ability to adapt to different microenvironments (75, 76), it is plausible to hypothesise that during PRRSV-1 infection, pulmonary macrophages undergo distinct activation phases. In the initial phase, macrophages undergo classic activation, also known as M1 polarisation, which is characterised by robust antimicrobial activity (77–79). After PRRSV-1 replication and PAMs cell death, there is an influx of monocytes and macrophages that replenish lung resident macrophages. These recruited cells would undergo a transition to an alternative activation phase, referred to as M2 polarisation as a consequence of the proinflammatory microenvironment induced by virulent PRRSV-1 strains in the lung. Although M2 macrophages are more susceptible to PRRSV infection (74, 80), these macrophages also exhibit anti-inflammatory properties and play a role in tissue repair and inflammation resolution (77–79).

### 3.2 Mechanisms involved in the regulation of lung inflammation

#### 3.2.1 Type I interferon, an interplay among IFN antiviral response and PRRSV replication

Type I interferons (IFNs), which include IFN-α, IFN-β, IFN-ε, IFN-ω, IFN-k, IFN-δ and IFN-τ, are essential for orchestrating effective antiviral innate and adaptive immune responses, restricting viral replication and viral spread (81, 82). Although PRRSV is highly susceptible to IFN-α both *in vitro* (62, 83, 84) and *in vivo* (85), it induces a weak or negligible production of type I IFN in PAMs and monocyte-derived dendritic cells (MoDCs) *in vitro*. However, systemic IFN-α has been detected following infection with various PRRSV isolates (26, 30, 85–89).

This finding suggests that specific cell types are engaged in sensing the infection, and the variation in IFN-α production could be attributed to strain-specific differences in IFN-α induction (30, 74). Notably, the virulent Lena strain, and probably other strains not studied in depth, increase in IFN-α mRNA in blood parallel to the viral load (30). A screening of IFN-stimulated genes (ISGs), a powerful instrument that interferes with viral replication, displayed an upregulation of these genes in bronchoalveolar lavage (BAL) cells from both virulent Lena- and moderately virulent 3249-infected piglets at 3 and 6 dpi (35). Interferon regulatory factors have been also found to be overexpressed in PAMs infected *in vitro* with Lena and LV strains (90). These findings add complexity to the immunopathogenesis of PRRSV infections, as IFN-α should serve as a trigger signal to the immune system and initiate the induction of adaptive immune responses, a process known to be inefficient during PRRSV infection in pigs (74, 91).

#### 3.2.2 Mechanisms of the proinflammatory response at lung level and its mirroring at systemic level

**Figure 8:**
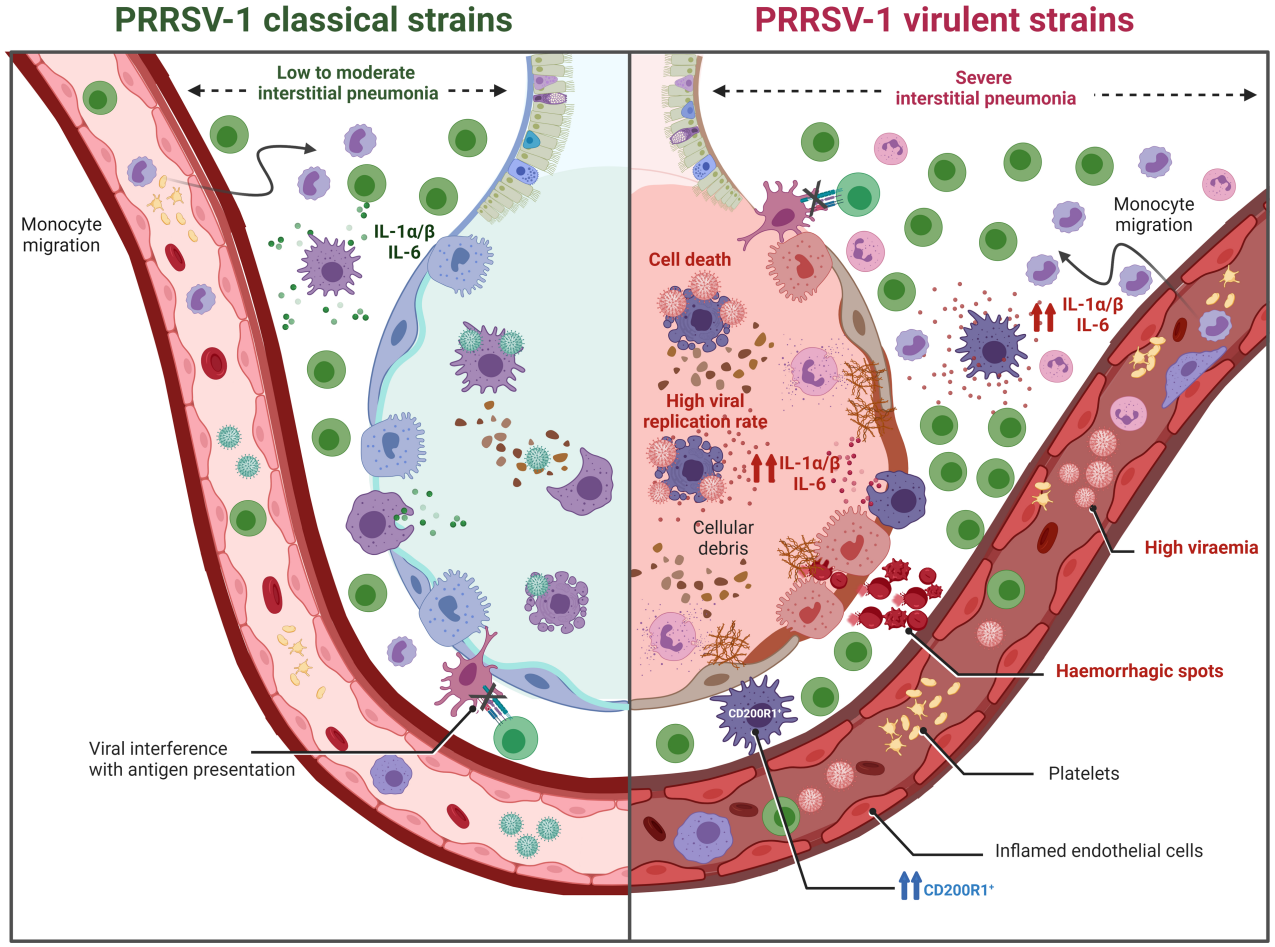
Graphic representation of pulmonary lesions induced by PRRSV-1 strains of different virulence (created with BioRender.com). PRRSV-1 classical strains (moderately virulent) typically induce a low to moderate interstitial pneumonia. In contrast, virulent PRRSV-1 strains exhibit heightened viral replication, leading to a significant reduction in pulmonary alveolar macrophages (PAMs) accompanied by early cell death of infected PAMs. Additionally, these strains trigger necrosis and apoptosis in other macrophages and lymphocytes, resulting not only in severe interstitial pneumonia but also, in some instances, in bronchitis, bronchiolitis, and bronchopneumonia. These alterations contribute to an imbalance in pulmonary immune homeostasis, making the host more susceptible to a wide spectrum of respiratory pathogens. The immunopathogenesis of these lesions is partly attributed to a stronger inflammatory response mediated by IL-1α/β when compared to low to moderately virulent strains.

The acute inflammatory response plays a crucial role in the host’s innate immune response. In piglets experimentally infected with moderately virulent PRRSV-1 strains, there is a local increase in the expression of IL-1α/β, IL-6, and TNF-α, which correlates with the development of interstitial pneumonia (Figure 8). Unlike other swine viruses, such as African swine fever virus (ASFV), SIV or PRCV, which induce a robust systemic inflammatory response, the serum levels of proinflammatory cytokines in PRRSV infection are limited (26, 53, 92, 93). Furthermore, the levels of these cytokines may vary depending on the PRRSV strain (94, 95). A recent *in vitro* model has reported that PRRSV-2 established similar infection landscapes in PIMs and PAMs but induced more acute and severe inflammatory responses and associated endothelial barrier damage in PIMs than PAMs. Additionally, the TNF-α and IL-1β induced by PRRSV infection disrupted the integrity of the endothelial barrier by dysregulating the tight junction proteins ocludin, claudin-1 and claudin-8, which might improve the permeability of pulmonary capillaries to further enhance the exchange of inflammatory substances and cells, ultimately promoting the development of interstitial pneumonia (96). These findings might be extrapolated to PRRSV-1 and suggest that while the lung tissue exhibits an inflammatory response primarily mediated by PIMs and interstitial macrophages, there is a lack of a systemic response. This phenomenon has been associated with a PRRSV strategy to evade the host’s immune response and promote viral persistence (61, 97).

Nevertheless, virulent PRRSV-1 strains are able to induce a strong activation of the immune system eliciting a robust systemic inflammatory response (Figure 8). This response is characterised by elevated levels of proinflammatory cytokines such as IL-1α/β, IL-6, TNF-α, or IFN-γ in the bloodstream, leading to high body temperature (ranging from 40.5°C to 42°C) and more severe and acute respiratory clinical signs and lesions. Virulent PRRSV-1 strains have demonstrated enhanced viral replication, resulting in a significant reduction of PAMs and an intensified inflammatory response, leading not only to severe interstitial pneumonia but also in some cases to bronchitis, bronchiolitis, and bronchopneumonia. The pathogenesis of these lesions is partly attributed to the inflammatory response mediated by IL-1α/β. It has been reported that virulent strains, such as Lena or SU1-bel, induced a higher expression of these proinflammatory cytokines compared to moderately virulent strains like LV or Belgium A (34, 38). Moreover, it is important to highlight the synergistic effect of co-infections between PRRSV-1 and bacteria, which triggers a cascade of proinflammatory cytokines that significantly intensify lung damage (98). Notably, not only virulent PRRSV-1 can upregulate the production of IL-1α/β but also some bacteria, such as *Glaesserella parasuis* or *Mycoplasma hyopneumoniae* (99, 100). This interaction between pathogens leads to more severe respiratory complications and exacerbate the overall disease outcome, particularly under field (natural) conditions where multiple pathogens may be present simultaneously.

Several mechanisms have been proposed to contribute to the increased severity of clinical signs and pulmonary lesions in PRRSV-1/bacteria co-infected animals. Firstly, the upregulation of CD14, the main receptor of the LBP (lipopolysaccharide-binding protein) complex. For instance, PRRSV-1 virulent strains, such as Lena, and Rosalía, along with other moderately virulent PRRSV-1 strains, induce the infiltration of CD14^+^ monocytes in the lungs, as well as PIMs and interstitial macrophages, which infiltrate extensive areas of the interstitium (71, 98, 99). While the influx of CD14^+^ immature macrophages and monocytes may represent an attempt to replenish the loss of CD163^+^ macrophages and restore the normal lung function, the increase in CD14^+^ cells also implies a higher availability of the LPS (lipopolysaccharide)-LBP complex receptor. This increased availability would predispose the lung to a higher production of proinflammatory cytokines upon exposure to bacterial LPS (98, 100–102).

The influence of the respiratory microbiota on the immune response to PRRSV-1 would be another mechanism involved in the increased severity of clinical signs and pulmonary lesions. Among the secondary bacteria isolated from PRRSV-1-infected pigs are low-virulent strains of *Actinobacillus pleuropneumoniae*, *Actinobacillus suis*, *Glaesserella parasuis*, *Pasteurella multocida*, and *Streptococcus suis*. These isolates are commonly associated with suppurative bronchopneumonia. The damage caused by PRRSV in the lung may create an imbalance in the respiratory microbiota, facilitating the growth and proliferation of these secondary bacterial infections, and leading to the development of more complex pneumonia processes, particularly in the case of virulent strains (69, 103, 104).

#### 3.2.3 Modulation and balance of the inflammatory response at lung level

Anti-inflammatory cytokines play an important role in immune homeostasis. Indeed, after a cascade of proinflammatory reactions and apoptosis in the lung, the host should be able to trigger the release of anti-inflammatory and/or regulatory mediators to limit the extent of the lung injury. During the acute phase of PRRSV-1 infection, an increase in CD200R1^+^ intravascular and interstitial macrophages and FoxP3^+^ cells have been associated with the severity of lung lesion, particularly within or surrounding foci of bronchopneumonia in piglets infected with the virulent Lena or 3249 PRRSV-1 strains (71). CD200R1 is known for its role in reducing the expression of proinflammatory cytokines in various inflammatory diseases (105). On the other side, FoxP3 is a marker of regulatory T cells (Tregs), which may act as inhibitor of the cell-mediated immune response in pigs upon PRRSV infection (106–109). Therefore, the upregulation of CD200R1^+^ and FoxP3^+^ cells represent potential mechanisms involved in the constraint and recovery of lung injury during acute PRRSV-1 infection together with the migration and replenishment of M2 macrophages to the lung.

Furthermore, recently, it has been published that PRRSV-1 may induce an imbalance between costimulatory and coinhibitory immune checkpoints at lung level during the acute phase of infection (110). Thus, it was reported that a modest increase in costimulatory molecules was accompanied by an earlier and more robust upregulation of coinhibitory molecules, particularly in the lungs of those infected with the virulent Lena strain (110). The concurrent expression of these coinhibitory immune checkpoints, as evidenced by the strong correlations observed among them, implies a synergistic action of these molecules, likely aimed at modulating the heightened inflammatory response and mitigating associated lung tissue damage.

The production of IL-10 would be another described mechanism involved in the resolution of inflammation during PRRSV infection. IL-10, a potent anti-inflammatory cytokine, can be induced by certain strains of PRRSV, including the more virulent ones. IL-10 not only controls tissue damage caused by the inflammatory response but also Th1 immune response. IL-10 induction can counteract the effects of IFN-γ and potentially stimulate the proliferation of Tregs. Some studies indicate that PRRSV infection leads to an increase in IL-10 levels, while others have not reported changes in the expression of this cytokine (107, 111–113). The lack of consensus among studies can be attributed to the fact that not all PRRSV strains induce IL-10 release (94, 95, 109, 112, 113).

## 4 Conclusion

This review delves into the macroscopic, microscopic, and molecular pathology induced by PRRSV-1 strains of different virulence in the lung, relating the different lesion and the molecular patterns. Although the hallmark lesion of interstitial pneumonia is always present in PRRSV infections, its temporal development, severity, and the possible occurrence of PNP and concurrent bronchopneumonia, are influenced by the virulence of the strain and the host-virus interactions. In addition, the way in which the survival and functionality of macrophage population is affected by the infection, and the mechanisms of activation and control of the inflammation occur, play a critical role in the manifestation of the disease. Differences in experimental settings and the emergence of new virulent strains make it difficult to draw a definitive picture of the immunopathogenesis of this disease, calling for the development of comparative experiments with the inclusion of reference strains.

## Author contributions

IR-T: Writing – review & editing, Writing – original draft. JS-C: Writing – review & editing, Writing – original draft. FS: Writing – review & editing, Writing – original draft, Conceptualization. FP: Writing – review & editing. LC: Writing – review & editing. EM: Writing – review & editing. JG-L: Writing – review & editing, Writing – original draft, Conceptualization. IMR-G: Writing – review & editing, Writing – original draft, Conceptualization.

## Glossary

PRRS: Porcine reproductive and respiratory syndrome
PRRSV: Porcine reproductive and respiratory syndrome virus
LV: Lelystad strain
PAM: Pulmonary alveolar macrophage
pi: Post-infection
dpi: Days post-infection
wpi: Weeks post-infection
PIM: Pulmonary intravascular macrophage
PCV2: Porcine circovirus type 2
SIV: Swine influenza virus
PRDC: Porcine respiratory disease complex
NETs: Neutrophil extracellular traps
PNP: Proliferative and necrotising pneumonia
PIM: Pulmonary intravascular macrophages
PRCV: Porcine respiratory coronavirus
MDMs: Monocyte-derived macrophages
IFNs: Type I interferons
MoDCs: Monocyte-derived dendritic cells
pDCs: Plasmacytoid dendritic cells
ISGs: IFN-stimulated genes
BAL: bronchoalveolar lavage
ASFV: African swine fever virus
LBP: Lipopolysaccharide-binding protein
LPS: Lipopolysaccharide
Tregs: Regulatory T cells
